# Post-COVID Factor X Deficiency: A Case Report From Pakistan

**DOI:** 10.7759/cureus.31473

**Published:** 2022-11-14

**Authors:** Omama Humayun, Talha Durrani, Rafiq Ullah, Ihtisham Qayum, Muhammad Ijaz Khan

**Affiliations:** 1 Internal Medicine, Khyber Medical College/Khyber Teaching Hospital, Peshawar, PAK; 2 Internal Medicine, Naas General Hospital, Naas, IRL

**Keywords:** factor x deficiency, acquired factor deficiency, clinical hematology, clotting disorder, covid 19

## Abstract

Acquired Factor X deficiency is a rare hematological disease, characterized by excessive bleeding, with fewer than 50 cases reported in the literature and practically all being associated with amyloidosis. We describe a case of a 38-year-old man with no known family history of hematologic disorders who had symptoms of a mild COVID-19 infection. Upon resolution, he developed excessive bleeding features, including epistaxis and hematuria. It was later found that while the rest of the coagulation factors were within normal limits, Factor X was 7% of the normal value, which reversed about two months after recovery. Our case highlights the significance of the less-expected post-COVID bleeding complications, in contrast to the classically seen thrombotic ones.

## Introduction

Coronavirus (COVID-19) infection is primarily a respiratory infection but it is associated with a host of hematological problems, including thrombocytopenia, disseminated intravascular coagulation (DIC), and coagulation factor deficiencies. Here, we present the first-ever reported case of post-COVID acquired Factor X deficiency (AFXD), a rare hematological disease, characterized by excessive bleeding.

## Case presentation

A 38-year-old male patient presented with cough, high-grade fever, and generalized body aches, with a high suspicion of COVID-19 infection which was confirmed positive on a polymerase chain reaction test. He was managed conservatively and recovered in three weeks, at which point he had a negative polymerase chain reaction test and a positive serum immunoglobulin G for COVID-19. One day later (the 22nd day of onset of COVID infection), he developed epistaxis, hematuria, hypogastric pain, and lower urinary tract symptoms (urgency, frequency, and weak stream). Of note, his family history was unremarkable for coagulopathy or hematological diseases. On examination, there was blood-stained mucus in the nares and minor gum bleeding, while bruises or rashes were essentially absent.

The baseline investigations including platelet count, D-dimer, bleeding time, hematocrit, kidney and liver function, and thyroid profile were within normal limits. His hepatitis B, hepatitis C, and human immunodeficiency virus serology were also negative. However, his hemoglobin was mildly low and the coagulation profile was grossly deranged with a prothrombin time of 60 seconds and activated partial thromboplastin time of 65 seconds. Consequently, correction studies were conducted that revealed improvement of prothrombin time from 59 to 16.5 seconds and activated partial thromboplastin time from 63 to 38 seconds. Furthermore, his urine analysis showed numerous red blood cells and pelvic sonography suggested a bladder clot, a seemingly paradoxical complication of gross hematuria. At this point, the presumed diagnosis of an acquired coagulopathy was made and a coagulation factor assay was sent, which revealed 7% activity of Factor X while other factors were reported within the reference range. Taken together, Factor X deficiency manifested by the patient appeared to be associated with the COVID-19 infection.

After admission to the General Medical Unit, he was administered intravenous vitamin K, multiple fresh frozen plasma transfusions, and passed urethral catheterization for continuous bladder wash with normal saline. He was also started on IV dexamethasone 16 mg once daily for five days and activated coagulation factors. As there was little improvement with the aforementioned treatment, plasma exchange (PLEX) therapy was commenced on alternate days, four sessions in total, during which his condition started to improve and his coagulation profile normalized. He was discharged on oral prednisolone for two weeks and after a one-month follow-up, his condition remained stable.

## Discussion

Herein, we report the first-ever case of AFXD following COVID-19 infection in an adult. AFXD is extremely rare and largely attributed to amyloidosis [1]. The second most common association is reported with a non-specific viral respiratory infection, i.e. non-amyloid AFXD [1,2], while its association with a prior COVID-19 has not been reported in the literature.

Our case denied any symptoms suggestive of myeloma or malignancy, exposure to toxins, or anticoagulation (warfarin, heparin, direct oral anticoagulants [DOACs]). Lee et al. 2012 [1] conducted a review of 34 cases of non-amyloid AFXD, reporting variability in the presenting features of AFXD from no or minor mucosal bleeding to lethal visceral hemorrhages regardless of the level of Factor X deficiency, with gastrointestinal bleeding being the most common (44%, n = 15), followed by hematuria [1,3]. A non-specific viral respiratory infection was found to be the most common association (38%, n = 13), preceding the manifestation of AFXD by one to four weeks suggesting that the non-amyloid AFXD may be a post-infectious sequel [1]. Thus, the present case is a typical presentation of non-amyloid AFXD.

Unlike thromboembolism in COVID-19, hemorrhagic events have been reported sporadically [4,5] and have largely been explained by DIC, thrombocytopenia, and anticoagulant overdose [6,7]. Among the hemorrhagic events thus mentioned, factor deficiencies have been rarely reported. These include factors V, VIII, XI, and XII but post-COVID Factor X deficiency has still not been reported in the literature [8-11].

Although the exact pathophysiology is uncertain, it is assumed that non-amyloid AFXD may be due to some transient inhibitors against Factor X [1,12,13]. In 1991, Mulhare et al. [12] demonstrated specific Factor X inhibitors in in vitro studies for the first time, postulating that antibodies against microorganisms cross-react with Factor X and Xa following an upper respiratory tract infection. Although suspected in several cases, they can be isolated in a few. In a review of 34 cases, specific Factor X inhibitors were reported in 26% (n = 9) cases [1]. In our case, mixing studies resulted in incomplete correction, and subsequently, Factor X deficiency was found on the coagulation factors assay, both raising the suspicion of a specific Factor X inhibitor. However, we did not investigate it due to financial constraints and low pre-test probability.

An array of treatment strategies is tried including fresh frozen plasma transfusions, vitamin K injections, recombinant Factor VII, prothrombin complex concentrate, activated coagulation factors, corticosteroids, intravenous immunoglobulin, and PLEX [2,3]. Plasma exchange, combined with steroid treatment and intravenous immunoglobulin, has been suggested as an effective strategy in non-amyloid AFXD [1]. Although we did not use prothrombin complex concentrate and rFVIIa because of affordability issues and the absence of life-threatening hemorrhage, our case improved dramatically with PLEX and corticosteroids.

In conclusion, AFXD has a proven association with a viral respiratory tract infection. In the absence of other underlying causes and the light of available literature, we can consider AFXD as post-infection sequelae to COVID-19 infection. In the evolving list of complications of COVID-19, as new data have to come to inform the expanding post-COVID sequel, this case adds to building data on it. This further helps clinicians and researchers to recognize, investigate, and manage bleeding complications in patients who have experienced a COVID-19 infection. 

## Conclusions

AFXD has a proven association with a viral respiratory tract infection; however, it has not yet been reported as a result of COVID-19. In the evolving list of complications of COVID-19, as new data have to come to inform the expanding post-COVID sequel, our case adds to building data on it. This further helps clinicians and researchers to recognize, investigate, and manage bleeding complications in patients who have experienced this infection. 
